# Vancomycin-laden calcium phosphate-calcium sulfate composite allows bone formation in a rat infection model

**DOI:** 10.1371/journal.pone.0222034

**Published:** 2019-09-19

**Authors:** K. Keely Boyle, Branden Sosa, Liza Osagie, Kathleen Turajane, Mathias P. G. Bostrom, Xu Yang

**Affiliations:** 1 University at Buffalo, Buffalo, New York, United States of America; 2 Hospital for Special Surgery, New York, New York, United States of America; 3 Royal Free Hospital, London, England, United Kingdom; Ohio State University, UNITED STATES

## Abstract

**Objective:**

Local antibiotic delivery systems with differing chemical and mechanical properties have been developed to assist in the management of osteomyelitis. We investigated the bone conductive and resorptive capabilities of a calcium phosphate-calcium sulfate (CaP/CaS) composite compared with commercially available polymethylmethacrylate (PMMA). In addition, we compared the *in vivo* preventative and treatment efficacies of both biomaterials in a proven osteomyelitis model.

**Methods:**

Sixty-four, male Sprague-Dawley rats were inoculated with 10 μl of 1.5 x 10^8^ CFU/ml of *Staphylococcus aureus* in a surgically drilled defect in the right proximal tibia. Infected animals were randomly allocated into prevention and treatment groups with 32 rats each. In the prevention group, the defect was filled with a plug containing either PMMA or CaP/CaS immediately after the inoculation. In the treatment group, the infected defects were irrigated, debrided, and filled with either a PMMA or CaP/CaS plug. Both CaP/CaS and PMMA were impregnated with 10% weight of vancomycin. Rats were sacrificed 6 weeks after cement insertion. Infection was detected by bacterial culture and histological analysis. Bone formation in the defect was assessed with micro-computed tomography and histology.

**Results:**

No bacteria were detected in any group. Both the prevention and treatment groups using CaP/CaS had significantly more bone volume fraction, bone area, and cartilage area than the PMMA groups.

**Conclusions:**

When loaded with 10% of vancomycin, CaP/CaS and PMMA have the same efficacy for treatment and prevention of osteomyelitis. CaP/CaS enhances bone defect healing through improved bone remodeling in our osteomyelitis rat model.

## Introduction

Infection after orthopaedic trauma, particularly osteomyelitis, is a devastating complication that delivers a significant challenge. The rate of infection after open fractures of long bones has been reported to be as high as 27%.[1] Successful treatment of osteomyelitis requires complete eradication of infection due to the high risk of recurrence, minimization of bone loss and restoration of biomechanical stability. Standard treatment protocol includes timely surgical debridement, appropriate systemic antibiotic therapy and dead space obliteration with the use of a local antibiotic delivery vehicle.[1–3] The use of antibiotic laden synthetic bone graft as bone void fillers has become common orthopaedic practice. Due to the limited availability of autologous bone graft and the various complications associated with allografts, utilizing synthetic bone graft is advantageous. (4) Synthetic grafting materials must exhibit a number of characteristics to maintain biological and mechanical stability, including having osteoconductive, osteoinductive and osteogenic properties. These features are critical in the setting of osteomyelitis associated with open fractures where substantial bone loss can occur.[4] Polymethylmethacralate (PMMA) has traditionally been the standard biomaterial facilitating localized antibiotic delivery and filler of bone defects, although there are disadvantages to its use.[5, 6] PMMA is not biodegradable and subsequent surgeries are often needed.[7] The cement usually needs to be removed after antibiotic elution with concomitant bone grafting procedures required for re-establishment of limb biomechanical stability.[8] Thus there is a substantial need for a rapidly ossifying, biodegradable scaffold with antibiotic eluding capabilities in the treatment of osteomyelitis.

Antibiotic impregnated biodegradable agents, such as calcium sulfate and calcium phosphate, have been developed to fill osseous defects in multiple infection related settings, including chronic implant-associated infection, infected nonunions, open fractures with associated bone loss and chronic osteomyelitis.[9–13] Calcium sulfate (CaS) demonstrates a number of characteristics important for maintaining biological and mechanical stability. Though not intrinsically osteoinductive, CaS has been shown to be an effective filler of osseous defects.[14] This material has demonstrated rapid resorption over six weeks, in which time neovascularisation occurs allowing osteoid deposition and the formation of new mineralized bone.[9, 13, 15, 16] This angiogenic effect and increased concentration of *in-situ* bone morphogenic proteins (BMPs), transcription growth factors and platelet-derived growth factors suggests that calcium sulfates play an active role in osteogenesis.[15, 17] Although the exact mechanism of formation remains unknown, there is a rapid and complete resorption without a localized inflammatory response.[18]

While the use of resorbable osteoconductive materials may eliminate the need for secondary procedures, the elution rates of antibiotics from such substances have proved variable.[6, 19, 20] Specifically, the rapid dissolution of CaS maybe too rapid while the use of a monolithic injectable calcium phosphate (CaP) may result in suboptimal prolonged retention of material.[19–21] Calcium phosphate-calcium sulfate (CaP/CaS) composites may provide a potential solution by combining rapid resorption of CaS with superior osteoconduction of CaP. Thus creating an injectable biphasic material with absolute resorption and improved bone induction capabilities while retaining antibiotic delivery capacity.

In this study, we used a rat osteomyelitis model previously developed by our group to investigate the bone formative and resorptive capabilities of the CaP/CaS composite in comparison to commercially available PMMA. (Heraeus Medical GmbH, Wehrheim, Germany) In addition, we made comparative *in vivo* analysis of the preventative and treatment efficacies of both biomaterials when impregnated with antibiotics.

## Materials and methods

### Preparation of tested materials

The CaP/CaS composite material, CERAMENT^™^ (Bone support, Lund, Sweden), used in this experiment is an injectable and moldable ceramic bone substitute intended for bone voids. The powdered composite is a 40% hydroxyapatite and 60% calcium sulfate composite, which was mixed manually with a radio contrast iohexol solution to create a workable paste. Contact with iohexol causes an ionization of the calcium sulfate hemihydrate, leading to its re-crystallization to calcium sulfate dihydrate; this crystalline structure forms a stable matrix through which the hydroxyapatite spreads. 2 grams of vancomycin powder (Hospira, Inc., Lake Forest, IL) was added to 20 grams of the dry composite, creating a 10% by weight cement material that was hand mixed until smooth. The concentration of antibiotics was based upon clinically relevant values and our previous *in vitro* studies.[22, 23] The paste was then spread into prefabricated (3 x 3 mm) molds and allowed to set to create uniform vancomycin-composite cylindrical plugs. Similarly, 2 grams of vancomycin was mixed with 20 grams Palacos^™^ PMMA cement (Heraeus Medical GmbH, Wehrheim, Germany) as per manufacturers guidelines. Using the same sized molds as used to make the composite plugs, 3 x 3 mm vancomycin-PMMA plugs were created. All the plugs were made immediately before the surgery.

### Animal model and study design

#### Anesthesia protocol

Using a protocol approved by the Institutional Animal Care and Use Committee of Hospital for Special Surgery, we randomly allocated 64 male Sprague-Dawley rats into a treatment and prevention arm (n = 32/arm) (Table 1). This study was implemented using an osteomyelitis model, which had been used by our group previously.[21] Isoflurane was administered to animals in chambers and once induced, they were given a combination of Ketamine (“Ketaset– 100 mg/mL) 75mg/kg and Xylazine (“Rompun” 20mg/mL) 10mg/kg. Ketamine and Xylazine were given intraperitoneally utilizing a 58”, 25 g needle and injecting into the right lower abdominal quadrant with the animal’s anterior body tilted down. This provided 15–20 minutes of surgical anesthesia and an isoflurane delivering nose cone was used for the remainder of the procedure.

**Table 1:** Study design.

	Treatment		Prevention	
	Cerament N = 16	PMMA N = 16	Cerament N = 16	PMMA N = 16
**0 Week**	Inoculation	Inoculation	Inoculation Cerament + Vancomycin	Inoculation PMMA + Vancomycin
**3 Week**	Irrigation & Debridement Cerament + Vancomycin	Irrigation & Debridement PMMA + Vancomycin		
**6 Week**			Sacrifice	Sacrifice
**9 week**	Sacrifice	Sacrifice		

### Surgical procedure

**Fig 1:**
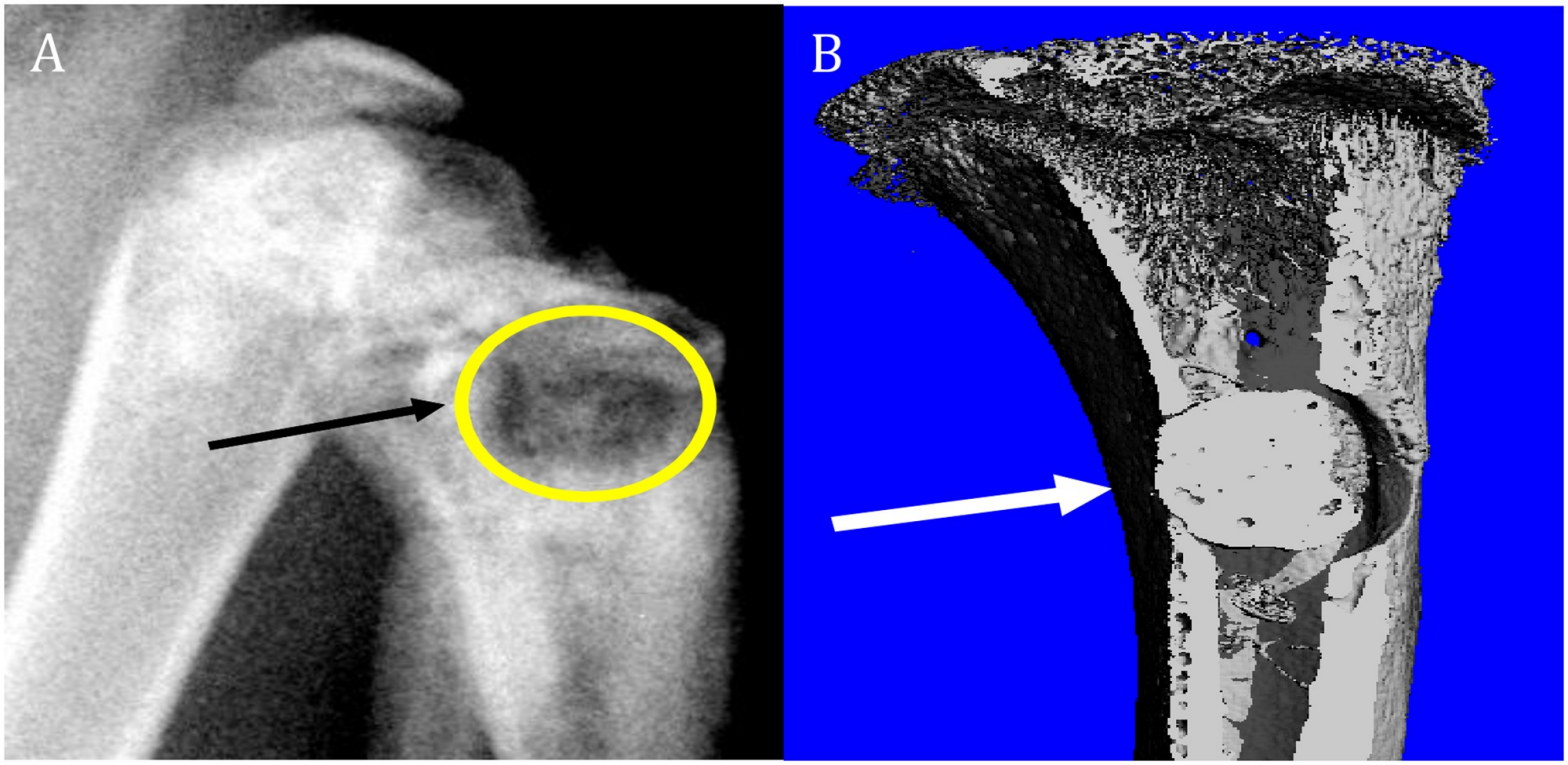
Radiological assessment. A) Lateral X-Ray image of right knee joint. The arrow and circle indicate the osteomyelitic lesion. B) MicroCT image of metaphyseal defect in the proximal tibia. The white arrow indicates the defect filled with PMMA.

After shaving and disinfecting the incision site with betadine and 70% ethanol, a 1 cm anteromedial incision over the right proximal tibia was made. A 3 mm in diameter and 3 mm in depth metaphyseal defect, 2 mm distal to the growth plate, was created using a Dynoics 450 drill (Smith & Nephew London, UK) (Fig 1). A single colony of methicillin-sensitive and vancomycin-sensitive *Staphyococcus aureus* ATCC 29213 obtained from our clinical microbiology laboratory was grown overnight in a shaking incubator, diluted to 0.5 McFarland Units (1.5 x 10^8^ CFU/ml), and 1.5x10^6^ CFU was inoculated in 10μl into the medullary canal through the defect to induce osteomyelitis.

**Fig 2:**
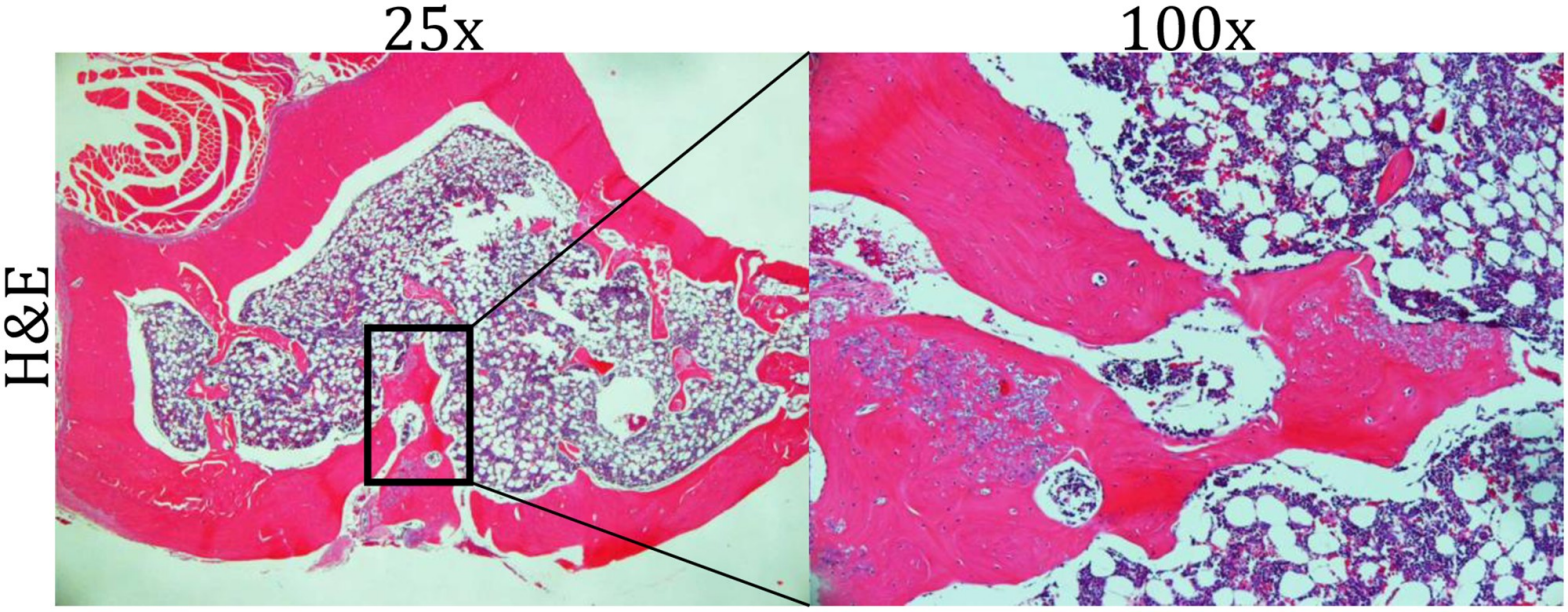
Histology of metaphyseal defect. Representative histological H&E axial section of rat tibia indicating no signs of infection, neutrophil infiltration, or osteolysis.

For the prevention arm, the defect was filled with either the CaS/CaP (n = 16) or PMMA (n = 16) plug immediately after the inoculation. For the treatment group, the defect was sealed by bone wax and bone infection was confirmed by high resolution x-ray (Faxitron X-ray Corp, Wheeling, IL) at 3 weeks after the inoculation (Fig 2). At this point, a second procedure was performed and there were clear macroscopic signs of osteomyelitis such as erythema in soft tissue, abscess, and bone destruction were verified. The defect was irrigated, debrided, and filled with either a CaS/CaP (n = 16) or PMMA (n = 16) plug. All rats were sacrificed 6 weeks following cement insertion.

After the procedure, rats were placed administered Sterile Lactated Ringers (10mL/kg/hr) subcutaneously (SC) and buprenorphine (Buprenex) 0.05mg/kg SC was given every 8–12 hours for 48 hours postoperatively. Rats were placed in clean cages and supplied with unrestricted access to irradiated trail mix and water. Animals were monitored daily for signs of systemic infection such as fever, dehydration, and dishevelled coat. After three weeks, infection was confirmed through an established infection radiographic scoring criteria. [24]

### Bacterial enumeration

To avoid contamination, all the procedures for tissue collection were carried out with standard sterile technique. The affected tibia (right) and surrounding muscle of all animals were harvested immediately after sacrifice. To establish a negative control, six contralateral limbs (left) were harvested at the same time as the affected limb. The drill hole was exposed and the tibia was cut transversely 5 mm distal to the infection site to expose the bone marrow to sonication. The samples were then placed in 10 mL of phosphate-buffered saline (PBS), vortexed for 20 seconds, sonicated for five minutes in a 20°C water bath, and vortexed again for 20 seconds. The tibias were then removed from the sonicate and placed in 10% of neutral buffered formalin for subsequent microCT and histological analysis. The sonicate was serially diluted and plated on 5% Sheep Blood, Tryptic Soy Agar. The plates were incubated at 37°C and the colonies were counted after 48 hours. Plates with 30–300 colonies were counted and multiplied by the respective dilution factor for the given plate, as per standard microbiological protocol.

### Microcomputed tomography (microCT)

After sonication, the right tibias were fixed in 10% formalin for 48 hours and scanned by microCT (Scanco Medical, Bassersdorf, Switzerland) with 20-μm isotropic voxel, 55kVp, 145μA, and 0.36° rotation step (180° angular range) per view. A global threshold of 417.0 mg HA [Hydroxyapetite] cm^3^ was used to segment mineralized tissue and effectively eliminate any signal from any residual CERMENT plug. Bone volume fraction (BV/TV) was measured in the area originally occupied by the pre-molded plug by using the software provided by the manufacturer. Bone volume (BV) was defined as the volume of new bone formed in the hole initially occupied by the CERAMENT or PMMA plug. Total volume (TV) was the volume of the hole. Bone volume fraction computed from microCT has been shown to accurately assess the strength and stiffness of cancellous bone.[25, 26]

### Histological analysis

After microCT scan, the samples were decalcified with 10% EDTA. PMMA plugs were removed from the bone. The specimens were then embedded with paraffin. Transverse, 7-μm sections of the proximal tibias were cut. Sections through the center of the defects were stained with Hematoxylin and Eosin (H&E) for gross morphology, silver to identify bacteria and alcian-blue to distinguish bone and cartilage. Images with 4X magnification were obtained (BIOQUANT Image Analysis Corp., Nashville, TN) to measure bone area and cartilage area (Image J v1.38, National Institutes of Health, Bethesda, Maryland, USA).

### Statistical analysis

Two-way ANOVA with Bonferroin post-hoc tests (SigmaStat Windows Version 2.03, SPSS Inc., Chicago, IL) was used to test the effect of cement (CaP/CaS or PMMA) and cement administration time (prevention of treatment) on BV/TV, bone area, and cartilage area. P < 0.05 was considered as significant.

## Results

Immediately postoperatively two animals in the PMMA and one in the CaP/CaS treatment groups died, two died due to pulmonary emboli at autopsy and the third due to anaesthetic complications. The remaining animals recovered well, maintaining consistent body weight in the post-inoculation period.

### Bone formation

**Fig 3:**
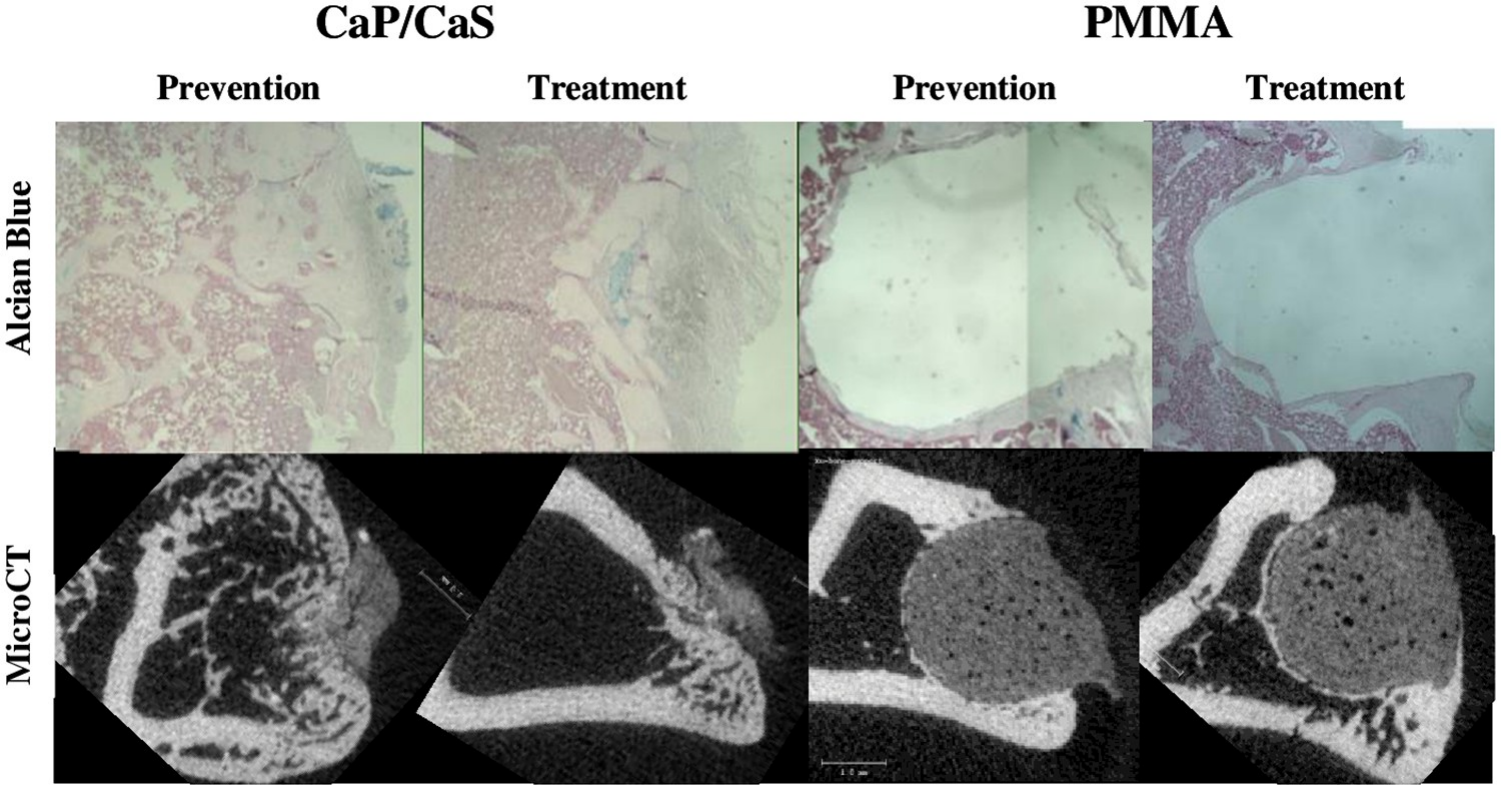
Alcian blue staining and microCT. Alcian blue staining and microCT analysis of the defect site exhibit plug resorption with CaP/CaS and preservation of the implantation space with PMMA in both prevention and treatment groups.

Based on microCT images, plugs were partially resorbed at 6 weeks in the CaP/CaS groups. New bone bridged the majority of the cortical defect. These were confirmed by histology with new bone and cartilage. In the PMMA groups, the size of the plugs did not change. Only a thin layer of new bone formed on the graft surface in the medullary canal. The defects could still clearly be observed (Fig 3).

**Fig 4:**
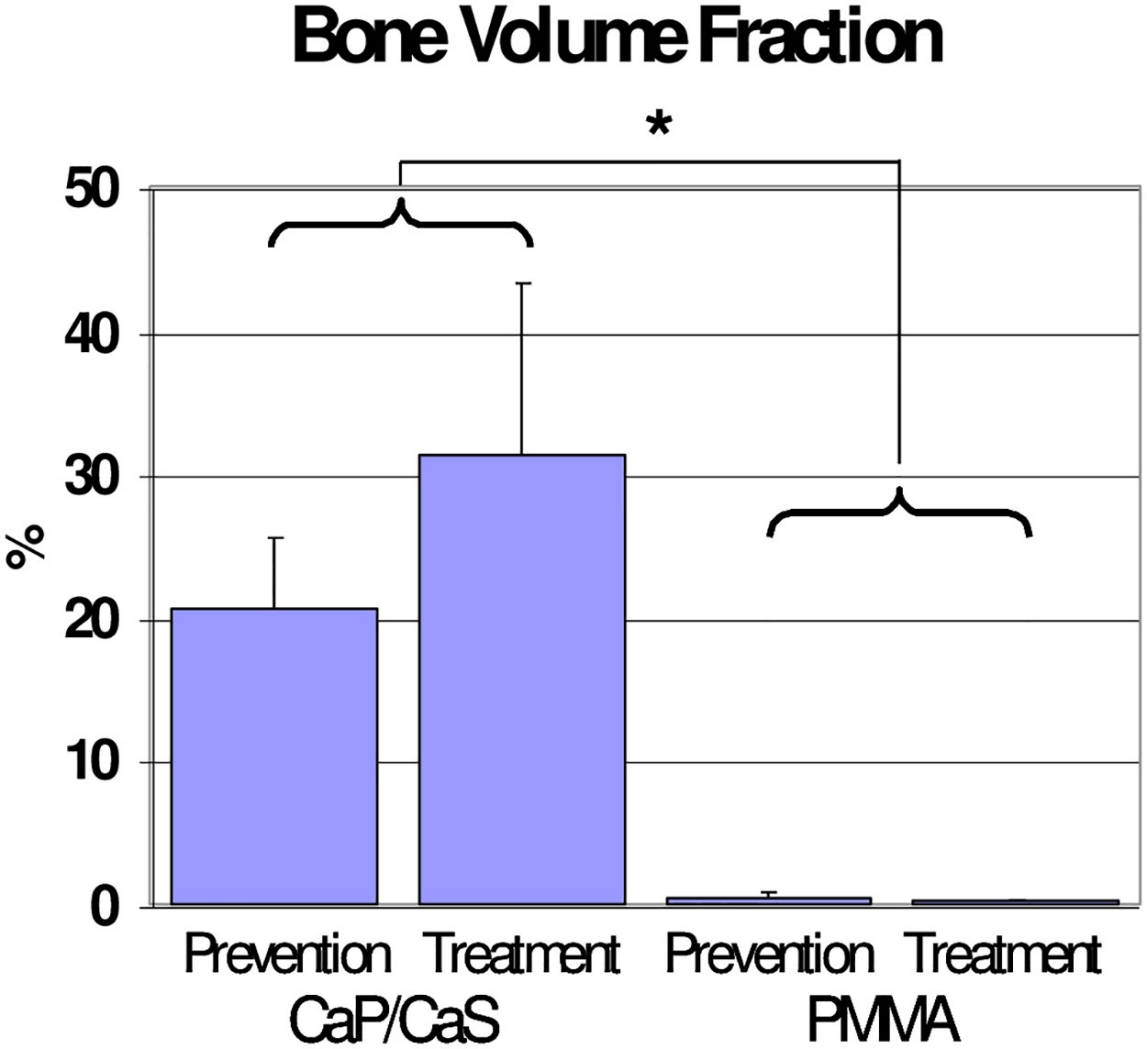
Bone volume fraction. MicroCT results reveal significant bone volume fraction at the site of plug implantation with CaP/CaS. Minimal bone volume fraction was observed with the use of PMMA. *: p < 0.05.

**Fig 5:**
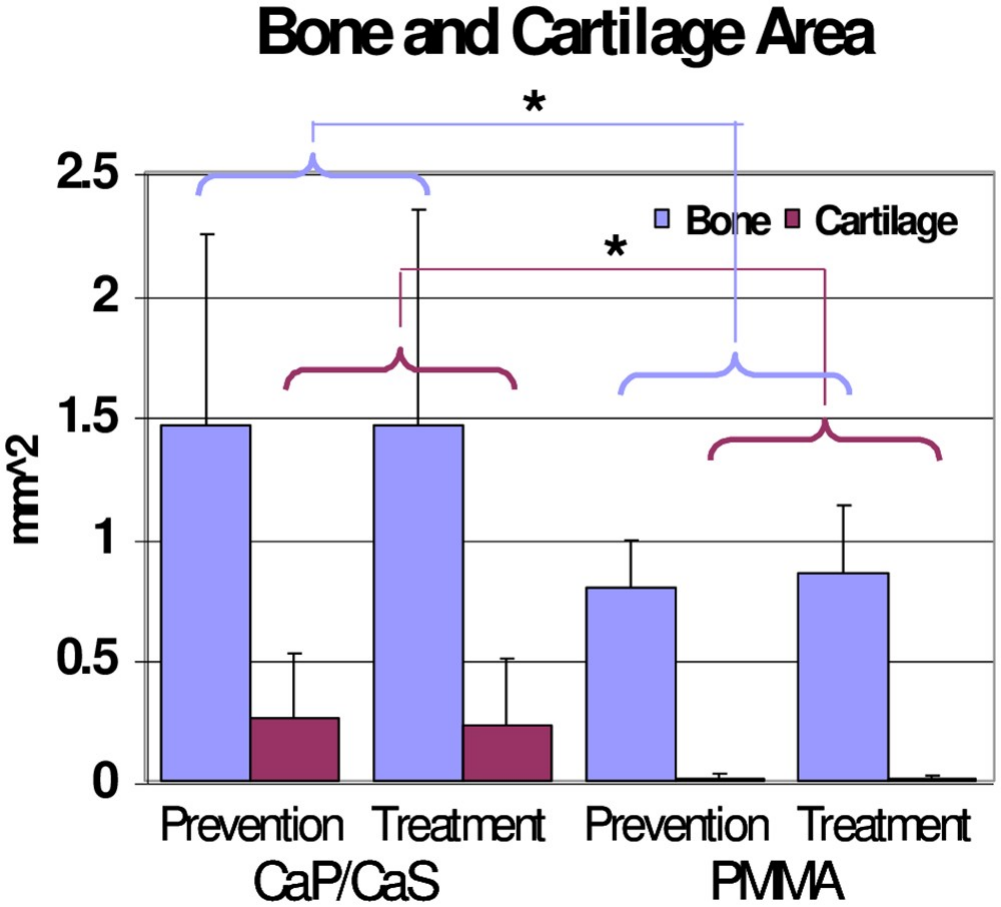
Bone and cartilage area. A significant increase in bone and cartilage area is observed with the use of CaP/CaS for prevention and treatment of osteomyelitis. *: p <0.05.

BV/TV was significantly higher in rats in the CaP/CaS groups than in the PMMA groups: 20.55% vs. 0.38% in the prevention arm and 31.27% vs. 0.15% in the treatment arm, respectively. (Fig 4). The same pattern showed in the histologic measures. In both the treatment and prevention arms, CaP/CaS increased bone area (1.46 vs. 0.80 mm^2^ in the prevention and 1.46 vs. 0.86 mm^2^ in the treatment arm) and cartilage area (0.26 vs. 0.01 mm^2^ in the prevention arm and 0.22 vs. 0.01 mm^2^ in the treatment arm) (Fig 5).

### Infection outcome

At dissection, no bony or soft tissue macroscopic signs of infection were noted. Similarly, after sonication of the proximal tibias no colony forming units of any organism were grown on agar plates, nor were any bacteria identified with silver staining. Histological examination showed no significant evidence of exudation or continued inflammatory tissue infiltration in any specimen (Fig 2). Based on these three methods of assessment, the bacterial infection had been eradicated in all cases. There was no evidence of persistent bone infection in any of the rats in both the treatment and prevention arms of the study at time of euthanasia.

## Discussion

The persistent and recurrent nature of osteomyelitis leads to significant challenges in successful eradication of established infection in bone. Efforts have focused on developing and optimizing management strategies for treating and preventing infection after open fracture.[1, 2, 9, 11, 27, 28] The widely accepted treatment for osteomyelitis incorporates thorough debridement of bone and soft tissues with systemic and local administration of antibiotics.[4, 29] The ability of biomaterials to locally deliver antibiotics has greatly contributed to the efficacious management of osteomyelitis, especially after surgical debridement where devitalized areas of bone have been removed and dead space is now present.

Acrylic based cements, such as PMMA, have traditionally been utilized as the biomaterial of choice to obliterate dead space from bone loss, although there are well-defined disadvantages.[2] Many surgeons have moved away from using PMMA in this setting and utilize various other bone void fillers, including other ceramics, allograft and synthetic bone graft. The inherent properties of PMMA and CaP/CaS differ substantially. PMMA was chosen for initial comparison to CaP/CaS for the ability to clearly visualize a histological delineation and try to quantify bone growth in CaP/CaS group. Prior studies evaluating antibiotic laden PMMA in the treatment of chronic osteomyelitis have shown variable elution rates, exothermic reactions and lack of biodegradability.[5, 22, 30] The inability for this material to be resorbed can cause a foreign body response in the host and has the potential to act as a reservoir for recurrent infection. The assiduous and invasive nature of osteomyelitis can be attributed to the bacterium’s ability to form biofilms.[31–33] These sessile microbial cells attach to a substratum, interface or each other in an organized manner where a multilayer structure can be produced.[34] Microorganisms embedded in a biofilm demonstrate impaired neutrophil-mediated killing and greater resistance patterns to antimicrobial agents.[35–37] Thus it is critical to understand the variable antibiotic elution characteristics, the possibility of subtherapeutic antimicrobial concentrations and optimal timing for removal of the cement.[22, 38] Previous studies with calcium sulphate alone have noted a variability in elution, with a marked decrease in concentration within the initial 72 hours; [39] whereas, CaP has been shown to have a prolonged elution rate of up to eight weeks.[20] Our previous *in vitro* work with this composite demonstrated that in combination, clinically significant vancomycin concentrations are eluted for up to 4 weeks.[21]

PMMA does not provide a scaffold or environment for bone to regrow and often requires autogenous bone grafting after removal.[2, 5, 8] The necessity of multiple procedures to eradicate the infection and re-establish biomechanical stability delays the healing process and imparts significant burden on patients. The results of this study using a resorbable calcium phosphate-calcium sulfate composite suggests a comparable antimicrobial delivery to that of PMMA, yet has the additional benefit of obliterating dead space and accelerating osteogenesis. The 20% increase in bone volume fraction observed in this study relates well to the clinical evidence existing for the use of calcium sulfate alone as a defect filler and graft expander.[9, 13] Prior work by Chang et al and Mckee et al demonstrated osteogenic efficacy of CaS alone in the setting of chronic osteomyelitis, revealing significantly improved healing rates following debridement and CaS implantation.[9, 40] These results suggest that the material resorbs at a similar rate to bone regeneration and is osteoconductive.[9] Blaha clearly outlined the osteoconductive capabilities of calcium sulfate, as well as its superior osteogenic activity in comparison to other bone substitutes in his 1998 review.[15] In this study, the combination of calcium sulfate with the biologically inert calcium phosphate resulted in a similarly osteoconductive material that retained the properties of the calcium phosphate, not just that of prolonged antibiotic elution, but also robust compressive strength associated with calcium phosphate based bone void fillers.

Our results demonstrated a comparable ability of the composite to eradicate infection to that of PMMA, yet with superior osteogenic and resorptive capabilities. The indirect bond within the composite allows calcium phosphate to spread evenly through the sulfate matrix. Consequently, *in vivo* calcium sulfate is resorbed, resulting in increased material porosity and allowing for bony ingrowth. Calcium phosphate is thought to remain stable and provide compressive strength throughout the ingrowth process. MicroCT imaging demonstrated guided bone formation both within and surrounding the resorbing composite pellet. This rapid restoration of bone is integral to the clinical management of dead space in the setting of osteomyelitis. The comparable results demonstrated in the preventative arm of the study reveal the plausible application of the composite in the treatment of polytraumatic open fractures, where bone loss and a high infection risk are concurrently problematic.

The decision to use water-soluble vancomycin is based upon its dose dependent nature. Local tissue levels may remain high after full cement elution, thus combating residual latent infection. The choice of antibiotic must take into consideration local microbial prevalence as well as the notion that high concentrations of certain antibiotics can affect the process of normal bone regeneration.[41] Although previous work has reviewed the treatment efficacies of tobramycin and gentamycin, it is thought that vancomycin is effective against the most common causative organisms, particularly methicillin resistant S*taphylococcus aureus* and other *Staphylococci* bacteria.[2, 9, 13, 14]

This study is subject to a number of limitations. The chronic and recurrent nature of osteomyelitis dictates our inability to fully assess the eradicative capabilities of either material fully. Vancomycin, in the absence of a study control, could have contributed to the outcome resulting in infection eradication. To truly relate to the clinical setting, animals would require monitoring for subtle systemic compromise both with haematogenous and biochemical markers. To fully appreciate the characteristics of the composite in comparison to PMMA, one should conduct mechanical testing to fully evaluate both bone strength and stiffness in and around the defect. Finally, this study is comparing a ceramic to an acrylic material. As such, the resorptive and osteoconductive characteristics differ. Future efforts will focus on comparing bone void filling materials with similar characteristics in an infection model that may be more relevant to current surgical techniques.

This work adds to the body of evidence supporting biodegradable alternatives to acrylic based cements. The CaP/CaS composite clearly provides advantageous properties as it is an efficacious vehicle for delivery of vancomycin and demonstrates a balance between biomaterial resorption and bone formation. This bone substitute assists in the prevention and treatment of osteomyelitis and its associated bony defects. The biodegradable, antibiotic eluding and osteogenic capabilities of this calcium phosphate-calcium sulfate composite allow for its application in a variety of clinical scenarios.

## Supporting information

S1 File Bone and cartilage area. (PDF)

S2 File Bone volume fraction. (PDF)

S3 File Bacterial counts. (PDF)
