# Supplementary material for: Vancomycin-laden calcium phosphate-calcium sulfate composite allows bone formation in a rat infection model
Source: PLoS One. 2019 Sep 19;14(9):e0222034. doi: 10.1371/journal.pone.0222034 (PMC6752756; doi:10.1371/journal.pone.0222034)
Supplement: S1 File — (PDF) [file pone.0222034.s001.pdf]

Treatment	Implant	Number	Bone Area	Cartilage Area
PRVT	Cerament	09R	1.115	0.376
PRVT	Cerament	11R	2.228	0.089
PRVT	Cerament	13R	0.968	0.057
PRVT	Cerament	15R	0.925	0.099
PRVT	Cerament	43R	3.681	0.140
PRVT	Cerament	47R	0.948	0.157
PRVT	Cerament	49R	1.249	0.570
PRVT	Cerament	51R	1.125	0.931
PRVT	Cerament	53R	1.246	0.160
PRVT	Cerament	55R	1.406	0.244
PRVT	Cerament	57R	1.272	0.000
PVRT	Cerament	59R	1.376	0.151
TRT	Cerament	01R	0.382	0.183
TRT	Cerament	06R	1.469	0.302
TRT	Cerament	07R	0.928	0.100
TRT	Cerament	21R	0.871	0.022
TRT	Cerament	25R	0.950	0.059
TRT	Cerament	27R	1.656	0.087
TRT	Cerament	29R	0.538	0.115
TRT	Cerament	31R	1.612	0.039
TRT	Cerament	33R	2.175	0.946
TRT	Cerament	35R	2.228	0.100
TRT	Cerament	39R	1.153	0.614
TRT	Cerament	41R	3.570	0.090
PRVT	PMMA	10R	0.945	0.000
PRVT	PMMA	12R	0.994	0.015
PRVT	PMMA	14R	0.905	0.000
PRVT	PMMA	16R	0.791	0.000
PRVT	PMMA	44R	0.280	0.004
PRVT	PMMA	46R	0.989	0.053
PRVT	PMMA	48R	0.561	0.016
PRVT	PMMA	50R	0.658	0.022
PRVT	PMMA	52R	1.026	0.031
PRVT	PMMA	54R	0.662	0.000
PRVT	PMMA	56R	0.726	0.000
PRVT	PMMA	58R	0.943	0.000
PRVT	PMMA	60R	0.747	0.010
PRVT	PMMA	62R	0.785	0.000
PRVT	PMMA	64R	0.850	0.049
TRT	PMMA	05R	0.724	0.000
TRT	PMMA	18R	1.026	0.000

TRT	PMMA	20R	0.539	0.000
TRT	PMMA	22R	0.619	0.000
TRT	PMMA	24R	1.263	0.020
TRT	PMMA	26R	0.838	0.000
TRT	PMMA	28R	0.976	0.000
TRT	PMMA	30R	1.025	0.018
TRT	PMMA	32R	0.548	0.000
TRT	PMMA	34R	1.232	0.000
TRT	PMMA	38R	1.078	0.032
TRT	PMMA	40R	0.906	0.000
TRT	PMMA	42R	0.377	0.000
