# Supplementary material for: Vancomycin-laden calcium phosphate-calcium sulfate composite allows bone formation in a rat infection model
Source: PLoS One. 2019 Sep 19;14(9):e0222034. doi: 10.1371/journal.pone.0222034 (PMC6752756; doi:10.1371/journal.pone.0222034)
Supplement: S2 File — (PDF) [file pone.0222034.s002.pdf]

BV/TV

Treatment	Implant	Number	BV/TV
PRVT	Cerament	09R	0.2776
PRVT	Cerament	11R	0.1882
PRVT	Cerament	13R	0.1112
PRVT	Cerament	15R	0.1998
PRVT	Cerament	43R	0.2053
PRVT	Cerament	47R	0.1466
PRVT	Cerament	49R	0.2299
PRVT	Cerament	51R	0.3120
PRVT	Cerament	53R	0.1647
PRVT	Cerament	55R	0.1988
PRVT	Cerament	57R	0.1886
PVRT	Cerament	59R	0.2330
PVRT	Cerament	61R	0.2121
PVRT	Cerament	63R	0.2088
TRT	Cerament	01R	0.4111
TRT	Cerament	06R	0.2644
TRT	Cerament	07R	0.1453
TRT	Cerament	17R	0.2810
TRT	Cerament	19R	0.2733
TRT	Cerament	21R	0.2100
TRT	Cerament	25R	0.2675
TRT	Cerament	27R	0.3179
TRT	Cerament	29R	0.2543
TRT	Cerament	31R	0.2699
TRT	Cerament	33R	0.3523
TRT	Cerament	35R	0.2770
TRT	Cerament	39R	0.4084
TRT	Cerament	41R	0.6453
PRVT	PMMA	10R	0.0043
PRVT	PMMA	12R	0.0009
PRVT	PMMA	14R	0.0014
PRVT	PMMA	16R	0.0034
PRVT	PMMA	44R	0.0007
PRVT	PMMA	46R	0.0014
PRVT	PMMA	48R	0.0166
PRVT	PMMA	50R	0.0048
PRVT	PMMA	52R	0.0031
PRVT	PMMA	54R	0.0024
PRVT	PMMA	56R	0.0020

PRVT	PMMA	58R	0.0021
PRVT	PMMA	62R	0.0089
PRVT	PMMA	64R	0.0017
PRVT	PMMA	02R	0.0005
TRT	PMMA	05R	0.0055
TRT	PMMA	18R	0.0001
TRT	PMMA	20R	0.0021
TRT	PMMA	22R	0.0017
TRT	PMMA	24R	0.0001
TRT	PMMA	26R	0.0000
TRT	PMMA	28R	0.0002
TRT	PMMA	30R	0.0024
TRT	PMMA	32R	0.0021
TRT	PMMA	34R	0.0008
TRT	PMMA	38R	0.0030
TRT	PMMA	40R	0.0019
TRT	PMMA	42R	0.0008
TRT	PMMA	43R	0.0006
