# Supplementary material for: Vancomycin-laden calcium phosphate-calcium sulfate composite allows bone formation in a rat infection model
Source: PLoS One. 2019 Sep 19;14(9):e0222034. doi: 10.1371/journal.pone.0222034 (PMC6752756; doi:10.1371/journal.pone.0222034)
Supplement: S3 File — (PDF) [file pone.0222034.s003.pdf]

Bacterial Counts

Treatment	Implant	Number	CFU
PRVT	Cerament	09R	0
PRVT	Cerament	11R	0
PRVT	Cerament	13R	0
PRVT	Cerament	15R	0
PRVT	Cerament	43R	0
PRVT	Cerament	47R	0
PRVT	Cerament	49R	0
PRVT	Cerament	51R	0
PRVT	Cerament	53R	0
PRVT	Cerament	55R	0
PRVT	Cerament	57R	0
PVRT	Cerament	59R	0
TRT	Cerament	01R	0
TRT	Cerament	06R	0
TRT	Cerament	07R	0
TRT	Cerament	21R	0
TRT	Cerament	25R	0
TRT	Cerament	27R	0
TRT	Cerament	29R	0
TRT	Cerament	31R	0
TRT	Cerament	33R	0
TRT	Cerament	35R	0
TRT	Cerament	39R	0
TRT	Cerament	41R	0
PRVT	PMMA	10R	0
PRVT	PMMA	12R	0
PRVT	PMMA	14R	0
PRVT	PMMA	16R	0
PRVT	PMMA	44R	0
PRVT	PMMA	46R	0
PRVT	PMMA	48R	0
PRVT	PMMA	50R	0
PRVT	PMMA	52R	0
PRVT	PMMA	54R	0
PRVT	PMMA	56R	0
PRVT	PMMA	58R	0
PRVT	PMMA	60R	0
PRVT	PMMA	62R	0
PRVT	PMMA	64R	0
TRT	PMMA	05R	0

TRT	PMMA	18R	0
TRT	PMMA	20R	0
TRT	PMMA	22R	0
TRT	PMMA	24R	0
TRT	PMMA	26R	0
TRT	PMMA	28R	0
TRT	PMMA	30R	0
TRT	PMMA	32R	0
TRT	PMMA	34R	0
TRT	PMMA	38R	0
TRT	PMMA	40R	0
TRT	PMMA	42R	0
